# Efficient Single-Photon Coupling from a Nitrogen-Vacancy Center Embedded in a Diamond Nanowire Utilizing an Optical Nanofiber

**DOI:** 10.1038/s41598-017-13309-z

**Published:** 2017-10-11

**Authors:** Yuya Yonezu, Kentaro Wakui, Kentaro Furusawa, Masahiro Takeoka, Kouichi Semba, Takao Aoki

**Affiliations:** 1 Department of Applied Physics, Waseda University, Okubo 3-4-1, Shinjuku, Tokyo, Japan; 2 National Institute of Information and Communications Technology (NICT), Nukui-kita 4-2-1, Koganei, Tokyo, Japan

## Abstract

Nitrogen-Vacancy (NV) centers in diamond are promising solid-state quantum emitters that can be utilized for photonic quantum applications. Various diamond nanophotonic devices have been fabricated for efficient extraction of single photons emitted from NV centers to a single guided mode. However, for constructing scalable quantum networks, further efficient coupling of single photons to a guided mode of a single-mode fiber (SMF) is indispensable and a difficult challenge. Here, we propose a novel efficient hybrid system between an optical nanofiber and a cylindrical-structured diamond nanowire. The maximum coupling efficiency as high as 75% for the sum of both fiber ends is obtained by numerical simulations. The proposed hybrid system will provide a simple and efficient interface between solid-state quantum emitters and a SMF suitable for constructing scalable quantum networks.

## Introduction

Solid-state quantum emitters, including semiconductor quantum dots and color centers in crystals, are indispensable elements in various quantum applications^1^. Among the solid-state quantum emitters, negatively-charged nitrogen-vacancy (NV) centers in diamond play a leading role due to their distinctive spin and optical properties, e.g. long coherence times of electron and nuclear spin states^2,3^, single-photon emission even at room temperature^4^, spin state initialization and read-out by incoherent excitation^5^. These properties of the NV centers are desirable for quantum information and sensing applications, such as quantum network^6,7^ and highly-sensitive quantum magnetometer^8–10^, and are also poteitially useful in quantum key distribution^11^ and linear optical quantum computation^12,13^.

One of the important requirements in these applications is an efficient coupling of the single-photons emitted from the NV centers, preferably into a guided mode of a single-mode fiber (SMF). In general, due to the high refractive index of diamond (*n*_d_ = 2.41), efficient extraction of single-photons from NV centers embedded in bulk diamond is challenging. A natural direction to overcome this problem is to use the diamond nanostructures, such as nanowires (nanopillars)^14–19^, photonic crystals^20–22^, and whispering-gallery-mode (WGM) disk resonators^23^, in which an NV center is embedded^14–17,20–22,24^, such that the spontaneous emission from the NV center to the cavity/wavegude mode is enhanced by Purcell effect^25^. However, in this approach, due to the large index mismatch between diamond and silica, it is challenging to efficiently couple the single-photons from the cavity/waveguide mode to the guided mode of the SMF.

The other possibility is to use the hybrid approach, where fiber-based nanophotonic devices are used as an interface to the diamond nanostructures, e.g. spherical diamond nanocrystals^26–32^ (see also refs^33–36^ for the related works) by taking advantages of evanescent coupling. As a fiber-based nanophotonic device, a nanofiber, a subwavelength-diameter region of a tapered optical fiber fabricated by heating and pulling a standard SMF^37–39^, has exclusively been employed thanks to the extended evanescent fields around the nanofiber that ensures good modal overlap with the diamond nanostuctures. The virtue of this type of the systems is that low-loss coupling to the SMF can be readily accomplished while making nanofiber itself albeit a very long taper is typically required in order to satisfy the adiabatic criterion. Thus, the interface issues to the NV centers could be effectively confined around the diamond nanostructures only, simplifying the device designs. Recently, such hybrid systems that consist of the nanofiber and the spherical diamond nanocrystal that contained a single NV center were reported both theoretically^28^ and experimentally^26,27,29^. For example, for the 100 nm-sized spherical nanocrystals, the maximum coupling efficiency to the two (backward and forward propagating) fiber modes was theoretically predicted to be 25%^28^. However, this value was rather limited by the poor mismatch between the emission patterns from the spherical nanocrystal and the guided mode within the nanofiber. This is similar to the hybrid system consisting of a nanofiber and a single atom^34^, which was experimentally confirmed by using a single colloidal quantum dot^35^.

To overcome this limitation, varieties of hybrid systems that contain structured emitters with nanofibers were discussed, including a semiconductor membrane^40,41^, a buried semiconductor waveguide^42,43^, and a linear photonic crystal cavity^44^. In these structured emitters, the emission from the NV is well coupled to one of the supported electromagnetic modes regardless of its orientation. More importantly, hybrid mode families are formed in these hybrid systems due to the coupling between electromagnetic modes originating from the isolated nanofiber and the structured emitter. At first glance, this means that the radiation losses induced by the butt coupling to the emitter structure (transition losses from the nanofiber to the hybrid system) may seriously impede the maximum coupling efficiency that can be achieved. Although dimples were typically introduced to nanofibers in order to avoid this type of losses, experimental uncertainties may also be increased^40–44^. In this regard, use of an adiabatically tapered diamond waveguide is promising^45–48^ and experimental realization of such a system was reported very recently^47^. The tapered diamond structures, fabricated by the top-down approach, i.e. e-beam lithography, can also readily be detached from the parent substrate using a micro-manipulator. Although the maximum coupling efficiency of 75% was predicted for such a system, experimentally observed coupling efficiency still remains to be 16–37%, possibly due to the difficulties in fabrication of the tapered diamond structures themselves.

Given these backgrounds, we numerically analyze rather a simpler hybrid system composed of a nanofiber and a cylindrical diamond nanowire that runs parallel to the nanofiber without any tapered structures. Note that high-quality cylindrical diamond structures containing negatively charged NV centers made of diamond crystals were already reported^14,16,17^ and thus the experimental implementation of such a system seems to be feasible. We found that even in such a simple system the coupling efficiency can be maximized as high as 75% by solely optimizing the system geometry. Our results highlight that the controls over the modal interference between the hybrid modes and the reflections from the nanowire end-facets are the important factors to achieve high coupling efficiencies while the transition losses at the end facets of the nanowire are negligibly small thanks to the extended evanescent fields of the hybrid modes to the surroundings. We also study the sensitivity of the system to the misalignment, showing that our proposed system exhibits reasonable device tolerances to potential fabrication errors. These results demonstrate a possibility of implementing a simple, but efficient interface between solid-state emitters and a SMF, which is useful for various quantum information applications and could be used as an important building block in scalable quantum networks.

## Results

### System description and supermode analyses

**Figure 1:**
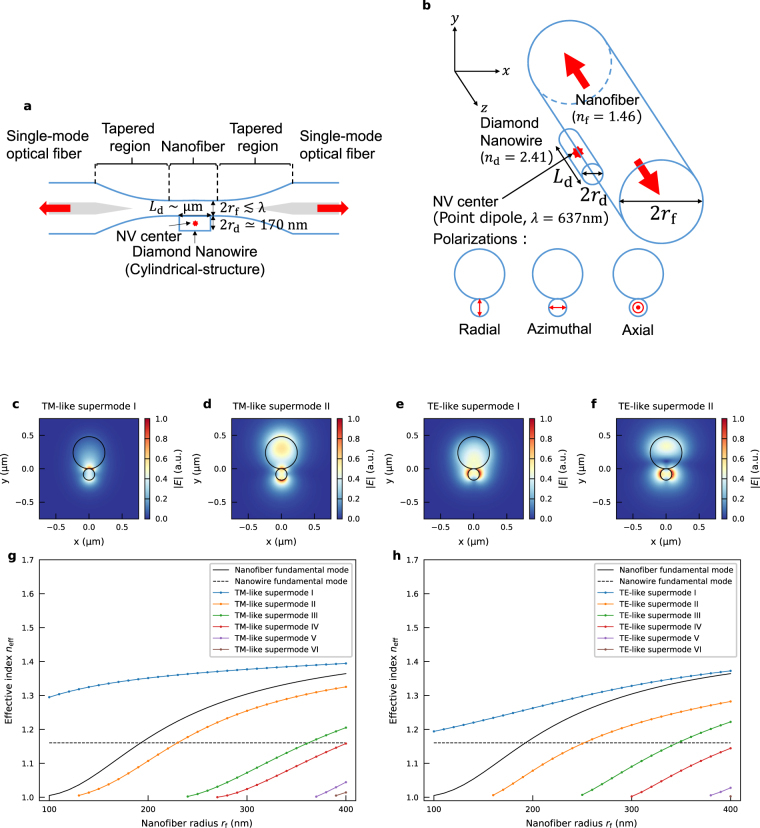
Coupling system between an optical nanofiber and a diamond nanowire. (**a**) Schematic of the proposed coupling system. (**b**) Geometry of numerical simulations. *r*_f_ is the radius of the nanofiber, *r*_d_ and *L*_d_ are the radius and the length of the diamond nanowire, respectively. *n*_f_ = 1.46 and *n*_d_ = 2.14 are the refractive indices of silica and diamond, respectively. (**c**–**f**) Electric field distributions of two principal supermodes for the case of the nanofiber radius *r*_f_ = 240 nm and the nanowire radius *r*_f_ = 85 nm. The black solid lines represent the surfaces of the nanofiber and the diamond nanowire. (**g**), (**h**) Effective indices as a function of the nanofiber radius *r*_f_ for TM-like supermodes and TE-like supermodes, respectively. The nanowire radius *r*_d_ = 85 nm is fixed. The black solid and short dashed lines represent the effective indices of the uncoupled nanofiber and diamond nanowire fundamental modes, respectively.

Figure 1a shows a schematic of our proposed system. A silica optical nanofiber with its radius of *r*_f_ is connected to SMFs via adiabatically tapered regions and is also in contact with a diamond nanowire with its radius of *r*_d_ and length of *L*_d_. An NV center, which we modelled as a point dipole oscillating at a wavelength of *λ* = 637 nm, is located at the center of the diamond nanowire. Once photons are coupled to the guided modes within the nanofiber, lossless propagation to the SMFs is assumed. Then, the nanofiber can be simplified as an infinite cylinder, as illustrated in Fig. 1b. The refractive indices of the silica and the diamond are assumed to be *n*_f_ = 1.46 and *n*_d_ = 2.41, respectively. In terms of dipole polarizaitons, we define them in a cartetian coordinate, but referred to, with respect to the center of the nanofiber, as radial (*y*), azimuthal (*x*), and axial (*z*).

Around the position of the dipole, supermodes originating from the respective structures (nanowire and nanofiber) are formed, which we refer to either nanowire-based or nanofiber-based supermode, respectively, and their spatial overlap with the radiation patterns of the dipole primarily governs the coupling efficiency to them. Therefore, we first conducted modal analyses for the supermodes by using a full vectorial finite-element method (FEM). Figure 1c–f show the electric field distributions of the four supermodes for the nanofiber radius of *r*_f_ = 240 nm and the nanowire radius of *r*_d_ = 85 nm. In all cases, the fields primarily concentrate around the surface of the diamond nanowire since the dimensions of the structures are small, as compared with the wavelength. Nevertheless, the orthogonality between the TE and TM-like modes are well maintained to evaluate the coupling efficiency. Therefore, it is assumed that the dipole emission from the radial and axial polarizations couples to the TM-like modes (Fig. 1c and d) whilst the one from the azimuthal polarization can only couple to the TE-like modes (Fig. 1e and f).

Figure 1g and h show the effective indices as a function of the nanofiber radius *r*_f_ for the TM-like and TE-like modes, respectively. The nanowire radius *r*_f_ = 85 nm is fixed. It was found that the system supports at most six supermodes for both polarization when the range of the nanofiber radius *r*_f_ is 100–400 nm at the wavelength of *λ* = 637 nm. The black solid and short dashed lines represent the effective indices of the fundamental modes in the respective structures (either nanofiber or nanowire alone). Note that the nanowire-based supermodes increase their effective indices as the nanofiber approaches to its proximity since it can be regarded that the effective cladding index is increased. By contrast, the effective indices of the nanofiber-based supermodes are reduced since the evanescent fields in the air is enhanced as the high index nanowire approaches. This indicates that the greatest field strength within the nanowire can be obtained by using the nanowire-based supermodes, to which the emission from the dipole preferentially couples.

### Optimization of the system geometry

**Figure 2:**
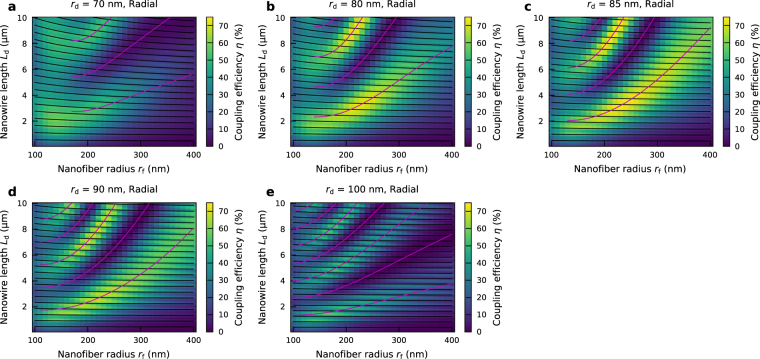
Dependence of the coupling efficiency on the system geometry. (**a**) Coupling efficiency as a function of the nanofiber radius *r*_f_ and the nanowire length *L*_d_ for the case of the nanowire radius *r*_d_ = 70 nm. (**b**–**e**) Same as (**a**), for the case of the nanowire radius *r*_d_ = 80 nm, 85 nm, 90 nm, and 100 nm, respectively. The magenta and black solid lines represent the beat length ${L}_{{\rm{d}},{\rm{beat}}}=\lambda /\{{n}_{{\rm{eff}},{\rm{s}}}^{\mathrm{(1)}}-{n}_{{\rm{eff}},{\rm{s}}}^{\mathrm{(2)}}\}$ and the Fabry-Perot resonant length ${L}_{{\rm{d}},{\rm{FP}}}=\lambda /{n}_{{\rm{eff}},{\rm{s}}}^{\mathrm{(1)}}$, respectively, obtained by the supermode analysis.

In addition to the modal overlap discussed above, the coupling efficiency also depends on the field distribution along the *z* axis due to the reflection from the nanowire end facets. To account for this effect, we performed calculations using three-dimensional finite-difference time-domain (3D-FDTD) method, where a point dipole embedded at the center of the nanowire is assumed as a source. The dipole polarization is tentatively fixed on the radial polarization in order to maximize the coupling efficiency. Figure 2a–e show the coupling efficiency as a function of the nanofiber radius *r*_f_ and the nanowire length *L*_d_ for different nanowire radii *r*_d_ = 70, 80, 85, 90, and 100 nm, respectively. The maximum coupling efficiency of 75% is obtained for the following parameters: nanofiber radius *r*_f_ = 240 nm, nanowire radius *r*_d_ = 85 nm, and the nanowire length *L*_d_ = 3.6 μm.

The results clearly show a long-range (~3–5 μm) periodic modulation along the nanowire length *L*_d_. This is attributed to the interference (or beating) between the two principal supermodes, as widely utilized in standard optical fiber couplers. At the center of the nanowire (*z* = 0), the optical power of the emission from the dipole is mainly coupled to the nanowire-based TM-like supermode due to the better modal overlap with the dipole emission pattern, as discussed. As this mode propagates towards the end facet of the nanowire ($z=\pm {L}_{{\rm{d}}}/2$), the power is gradually transferred to the nanofiber-based supermode with the same polarization. The maximum power transfer occurs when the accumulated phase difference between these two supermodes becomes an odd multiple of *π*. This gives rise to a periodic modulation of the coupling efficiency as a function of *L*_d_. This beat period is determined by the difference of the effective indices between the two supermodes as ${L}_{{\rm{d}},{\rm{beat}}}=\lambda /\{{n}_{{\rm{eff}},{\rm{s}}}^{\mathrm{(1)}}-{n}_{{\rm{eff}},{\rm{s}}}^{\mathrm{(2)}}\}$, where ${n}_{{\rm{eff}},{\rm{s}}}^{\mathrm{(1)}}$ and ${n}_{{\rm{eff}},{\rm{s}}}^{\mathrm{(2)}}$ are the effective refractive indices of the nanowire-based and nanofiber-based supermodes, respectively, and agrees well with the modal analyses given in the previous section (see the magenta solid lines in the figures). Although the other high order modes are supported within the structures for ${r}_{{\rm{f}}}\,\gtrsim$ 250 nm, the interference related to those modes are not noticeable possibly because of the large difference in the effective indices that limits the achievable amount of power transfer from the nanowire-based supermode.

Besides, a short-range (~500 nm) periodic oscillation is also superimposed in Fig. 2a–e (the black solid lines). This can be attributed to the Fabry-Perot effect caused by the reflection at the nanowire end facets, which in turn modifies the electric fields around the position of the dipole due to the formation of standing waves within the nanowire. Since this period agrees with ${L}_{{\rm{d}},{\rm{FP}}}=\lambda /{n}_{{\rm{eff}},{\rm{s}}}^{\mathrm{(1)}}$, the assumption that the power coupling from the dipole is dominated by the nanowire-based supermode is also validated.

### Dependence on the dipole polarizations

Towards experimental demonstration of the proposed system, one of the important factors is the dipole polarizations. In the above calculation, the dipole polarization is fixed on the radial polarization. However, from an experimental point of view, the alignment of the dipole polarization is challenging. In addition, a real NV center has two orthogonal dipoles in the plane perpendicular to the NV axis. Therefore, in order to evaluate the coupling efficiency, the emission from these dipoles should be considered as a superposition of the emission of the three dipole polarizations (radial, azimuthal, and axial)^28^.

**Figure 3:**
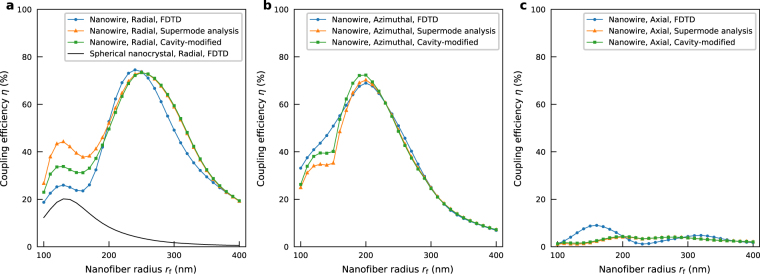
Dependence of the coupling efficiency on the dipole polarizations. (**a**) Radial polarization. (**b**) Azimuthal polarization. (**c**) Axial polarization. The circles, triangles, and squares represent the FDTD, supermode analysis (based on the model in ref.^40^), and additional cavity-modified (based on the model in ref.^57^) results, respectively. The nanowire radius *r*_d_ = 85 nm and the nanowire length *L*_d_ = 3.6 μm are fixed. For comparison, the black solid line in Fig. 3a represents the FDTD result for the case of the spherical diamond nanocrystal (the radius *r*_nc_ = 85 nm) with radial polarization.

Figure 3a–c shows the dependence of the coupling efficiency on the dipole polarizations. The nanowire radius *r*_d_ = 85 nm and the nanowire length *L*_d_ = 3.6 μm are fixed. The dipole is embedded in the center of the nanowire. For the case of the radial polarization (included in Fig. 2c), the maximum coupling efficiency of 75% at the nanofiber radius *r*_f_ = 240 nm is higher than those achieved by both the azimuthal and axial polarizations cases. For the case of the azimuthal polarization, the maximum coupling efficiency of 69% is still obtained at the nanofiber radius *r*_f_ = 200 nm. For the case of the axial polarization, the maximum coupling efficiency of only 9% is obtained at the nanofiber radius *r*_f_ = 160 nm, which is due to the fact that the axial component of the electric fields of the supermodes is small in comparison with the radial or azimuthal components. These results show that selecting the radial or azimuthal polarizations is indispensable in order to obtain the high coupling efficiency. Note that recent remarkable progress of NV-axis-alignment techniques^49^ may enable us to select the radial or azimuthal polarizations before the fabrication of the diamond nanowires. For comparison, the black solid line in Fig. 3a shows the FDTD result for the case of the spherical diamond nanocrystal (the radius *r*_nc_ = 85 nm) with the radial polarization. The maximum coupling efficiency of 20% is obtained at the nanofiber radius *r*_f_ = 130 nm, similarly to the previous report^28^. The efficiency peak is obtained with thicker nanofiber radius in our system (*r*_f_ = 240 nm) than the spherical nanocrystal case (*r*_f_ = 130 nm), and which is advantageous from a practical point of view since the length of the tapers to the nanofiber can be shortened.

Although the 3D-FDTD method is a powerful tool for investigating properties of electromagnetic fields in relatively complicated optical components, such as composite waveguide systems, it does not always provide enough information to explain the physical effects. Thus, in order to validate the 3D-FDTD calculations above, we calculate the coupling efficiency by the supermode analysis based on the model in ref.^40^. The triangles in Fig. 3a–c show the coupling efficiency obtained by the supermode analysis. When the nanofiber radius *r*_f_ is large (${r}_{{\rm{f}}}\,\gtrsim$ 200 nm), the 3D-FDTD results can be reproduced well by the supermode analysis. However, when the nanofiber radius *r*_f_ is small (${r}_{{\rm{f}}}\,\lesssim$ 200 nm), the results obtained by the two methods are markedly different.

The main cause of the difference is considered to be a lack of the contribution of the Fabry-Perot resonance due to the reflection from the nanowire facets in the above supermode analysis. When the nanofiber radius *r*_f_ becomes small with the constant nanowire radius *r*_d_, the effect of the nanowire facets becomes relatively large. The squares in Fig. 3a–c show the results obtained by the additional modification, where the reflection of only the nanowire-based supermode from the diamond nanowire facets was taken into account. The modified supermode analysis results clearly approach the 3D-FDTD results. Note that the modified supermode analysis could be further improved by using more accurate estimation of the reflection coefficients of the multi-mode nanowire^50^.

### Dependence on the dipole positions

**Figure 4:**
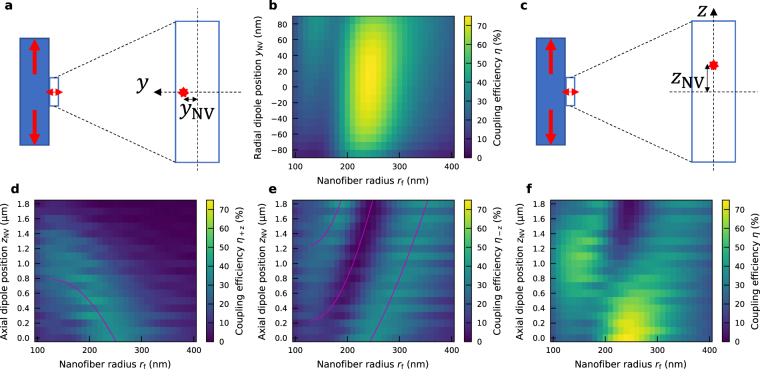
Dependence of the coupling efficiency on the dipole positions. (**a**) Schematic of the radial dipole position *y*_NV_ in the diamond nanowire. (**b**) Dependence of the coupling efficiency on the radial dipole position *y*_NV_ from the radial center of the diamond nanowire and the nanofiber radius *r*_f_ for the case of the nanowire radius *r*_d_ = 85 nm and the nanowire length *L*_d_ = 3.6 μm with radial polarization. (**c**) Schematic of the axial dipole position *z*_NV_ in the diamond nanowire. (**d**–**f**) Dependence of the coupling efficiency on the axial dipole position *z*_NV_ from the axial center of the diamond nanowire to the +*z* direction and the nanofiber radius *r*_f_ for the same geometry as that of (**b**). (**d**), (**e**), and (**f**) represent the coupling efficiencies for +*z* fiber end, −*z* fiber end, and the sum of both fiber ends, respectively. The magenta solid lines in (**d**) and (**e**) represent the beat effect against the change of the axial dipole position *z*_NV_ for the +z and −*z* directions, respectively.

Another issue is the dipole position in the nanowire. Figure 4a shows a schematic of the radial dipole position *y*_NV_ in the diamond nanowire. Figure 4b shows the dependence of the coupling efficiency on the radial dipole position *y*_NV_. The nanowire radius *r*_d_ = 85 nm and the nanowire length *L*_d_ = 3.6 μm are fixed. The dipole polarization is radial polarization. For all the radial dipole positions *y*_NV_ between −35 nm and 55 nm, the maximum coupling efficiencies of higher than 70% are obtained. Such insensitivity to the change of the radial dipole position *y*_NV_ is reflected by the fact that the electric fields of the supermodes in the nanowire are moderately changed against the radial direction^36^.

Figure 4c shows a schematic of the axial dipole position *z*_NV_ in the diamond nanowire. Figure 4d–f show the dependence of the coupling efficiency for +*z* direction, −*z* direction, and the sum of both fiber ends, respectively, on the axial dipole position *z*_NV_. The nanowire radius *r*_d_, the nanowire length *L*_d_ and the dipole polarization are the same as those used in Fig. 4b. The dependence of the coupling efficiency for the ±*z* directions on the axial dipole position *z*_NV_ can be explained by the beat effect. Since the nanowire length *L*_d_ is fixed, the distance between the dipole and the nanowire facets decreases (increases) for the +*z* (−*z*) direction. Therefore, the peak nanofiber radius *r*_f_, with which the maximum power transfer occurs from the nanowire-based supermode to the nanofiber-based one, for the +*z* (−*z*) direction decreases (increases) as shown in Fig. 4d(e). These trends can be quantitatively assessed by calculating ${z}_{{\rm{NV}},{\rm{beat}}}=\pm \{{L}_{{\rm{d}}}-m{L}_{{\rm{d}},{\rm{beat}}}({r}_{{\rm{f}}})\}/2\,(m=\mathrm{1,}\,\mathrm{2,}\,\mathrm{...)}$, as indicated by the magenta solid lines. Consequently, when the axial dipole position *z*_NV_ is less than 0.2 μm, the maximum coupling efficiencies higher than 70% are obtained. This requirement for the positioning accuracy could serve as a benchmark value for fabrication, and can well be achieved by utilizing the recent accurate NV-positioning technologies (e.g. focused electron irradiation^51^ or laser writing^52^) after the nanowire fabrication.

### Dependence on the orientation of the nanowire

**Figure 5:**
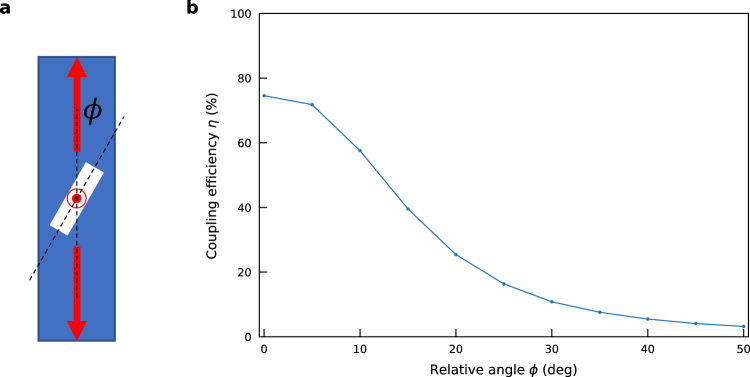
Dependence of the coupling efficiency on the orientation of the nanowire. (**a**) Schematic of the relative angle *ϕ* between the nanofiber and the diamond nanowire. (**b**) Dependence of the coupling efficiency on the relative angle *ϕ* for the case of the nanofiber radius ${r}_{{\rm{f}}}\,=$ 240 nm, the diamond nanowire radius ${r}_{{\rm{d}}}\,=$ 85 nm, and the diamond nanowire length ${L}_{{\rm{d}}}\,=$ 3.6 μm with radial polarization.

Finally, we briefly discuss about the alignment of the nanowire to the direction of the nanofiber. Figure 5a shows a schematic of the relative angle *ϕ* between the nanofiber and the diamond nanowire. The nanowire radius *r*_d_ = 85 nm, the nanowire length *L*_d_ = 3.6 μm, and the nanofiber radius *r*_f_ = 240 nm are fixed again. The dipole is embedded in the center of the nanowire with the radial polarization. Figure 5b shows the dependence of the coupling efficiency on the relative angle *ϕ*. When the relative angle *ϕ* becomes larger than 20°, the coupling efficiency monotonously decreases below 25% (comparable to the spherical nanocrystal case). Therefore, a precise positioning of the nanowire utilizing nanowire manipulation techniques, such as atomic force microscopy (AFM) manipulation^53,54^ and optical tweezers^55,56^, is indispensable towards the experimental realization of the proposed system. Note that a deterministic pick-and-place operation of a 12 μm long diamond waveguide on the nanofiber with the radius of 250 nm by using a tungsten micro-manipulator tip has been demonstrated^47^, which implies that alignment errors less than ~2.4° can be achieved by using such a technique with realistic conditions. Therefore, it should be emphasized that the maximum coupling efficiencies of higher than 70% could be obtained, when the relative angle *ϕ* is less than 5° as shown in Fig. 5b.

## Conclusion

In conclusion, we have proposed a novel efficient hybrid system between the nanofiber and the diamond nanowire. A main advantage of our proposed coupling system is that the fabrication of the diamond nanowire could be easier than the other techniques, e.g. the adiabatically tapered diamond waveguide^47,48^. Assuming a cylindrical diamond nanowire with flat facets (i.e. no tapered-structures), the maximum coupling efficiency as high as 75% for the sum of both fiber ends is numerically obtained by optimizing the system geometry (the nanofiber radius *r*_f_ = 240 nm, the nanowire radius *r*_d_ = 85 nm, and the nanowire length *L*_d_ = 3.6 μm). Our numerical results indicate that the optimization of the two physical effects are important to maximize the coupling efficiency: (1) the interference between the two supermodes and (2) the Fabry-Perot resonance due to the reflection from the nanowire facets.

To investigate the experimental feasibility, we evaluated the dependences of the coupling efficiency on the dipole polarizations, the dipole positions, and the orientation of the nanowire, respectively. Our numerical results show that, in order to obtain high coupling efficiency, (1) the NV axis should be aligned parallel to the nanowire axis, (2) the NV center (dipole) should be placed at the center of the nanowire within ±0.2 *μ*m in the axial direction, and (3) the alignment tolerance of the nanowire orientation to the nanofiber should be ±5°. These requirements could be fulfilled by taking advantages of recent progress on the diamond nano-fabrication technologies, such as the NV-axis-alignment technique^49^ to control the dipole polarization, the NV-positioning techiques^51,52^, and the nano-manipulation techniques^47,53–56^ for the precise alignment.

Our proposed coupling system will provide a simple and efficient interface between solid-state quantum emitters and a SMF suitable for constructing scalable quantum networks. In addition, although we have paid attention to only NV centers in diamond so far, our system can be also applied to the other solid-state quantum emitters, such as the other color centers in diamond (e.g. SiV centers^48^) and semiconductor quantum dots^54^, embedded in the nanowire. Moreover, for the further improvement of the coupling efficiency, our system can be easily combined with the nanofiber-based resonators^57–60^.

## Methods

### 3D-FDTD simulation

The three-dimensional finite-difference time-domain (3D-FDTD) method (FDTD solutions, Lumerical) is used for the numerical simulations. The nanofiber and the diamond nanowire are assumed as a cylindrical-structured silica (refractive index *n*_f_ = 1.46) with the radius *r*_f_ and a cylindrical-structured diamond (refractive index *n*_d_ = 2.41) with the radius *r*_d_ and the length *L*_d_, respectively. We calculate the coupling efficiency of the radiation from a point dipole source (a simplified model of a single NV center, *λ* = 637 nm) embedded in the diamond nanowire placed on the surface of the nanofiber to the fundamental guided mode of the nanofiber for the sum of both fiber ends, with the geometry as shown in Fig. 1b. The computational domain is a box of 3 μm × 3 μm × 15 μm, which is surrounded by perfectly matched layers (PMLs). Note that the computational domain is reduced by half, taking advantage of the system symmetry: either a perfect magnetic or electric wall is placed to the *yz* plane, except for the calculation of the dependence on the orientation of the nanowire. We use non-uniform mesh sizes, which are automatically optimized by the FDTD software depending on the simulation geometry, e.g. nanofiber/nanowire sizes. (The typical transverse mesh sizes around the interface between the nanofiber and the nanowire are smaller than 5 nm, which is the minimum step used to change the nanowire radius *r*_d_.)

### Supermode analysis

The full vectorial finite-element method (FEM, Comsol Multiphysics) is used for the supermode analysis based on the model in ref.^40^. Computational domain is also reduced by half. The coupling efficiency is determined by the spontaneous emission rates of a two-level atom into the supermodes derived from the Heisenberg equations. In order to investigate the contribution of the Fabry-Perot resonance due to the reflection from the nanowire facets, we additionally modify the calculations by using the cavity-modified spontaneous emission rates based on the model in ref.^57^. For simplicity, we assume that the rate of the spontaneous emission coupled to only the nanowire-based supermode is modified by the reflection from the diamond nanowire facets with the approximate reflection coefficient ${r}_{1}=({n}_{{\rm{eff}},{\rm{s}}}^{\mathrm{(1)}}-{n}_{{\rm{eff}},{\rm{f}}}^{\mathrm{(1)}})/({n}_{{\rm{eff}},{\rm{s}}}^{\mathrm{(1)}}+{n}_{{\rm{eff}},{\rm{f}}}^{\mathrm{(1)}})$, where ${n}_{{\rm{eff}},{\rm{s}}}^{\mathrm{(1)}}$ and ${n}_{{\rm{eff}},{\rm{f}}}^{\mathrm{(1)}}$ are the effective refractive indices of the nanowire-based supermode and the fundamental guided mode of the nanofiber, respectively.
